# Stress-Related Mental Health Disorders and Inflammation in Pregnancy: The Current Landscape and the Need for Further Investigation

**DOI:** 10.3389/fpsyt.2022.868936

**Published:** 2022-06-28

**Authors:** Meghna Ravi, Brandy Bernabe, Vasiliki Michopoulos

**Affiliations:** ^1^Graduate Division of Biological and Biomedical Sciences, Laney Graduate School, Emory University, Atlanta, GA, United States; ^2^Department of Psychiatry and Behavioral Sciences, Emory University School of Medicine, Atlanta, GA, United States; ^3^Emory National Primate Research Center, Atlanta, GA, United States

**Keywords:** inflammation, pregnancy, stress, mental health, women's health

## Abstract

Many studies have focused on psychoimmunological mechanisms of risk for stress-related mental health disorders. However, significantly fewer studies have focused on understanding mechanisms of risk for stress-related disorders during pregnancy, a period characterized by dramatic changes in both the innate and adaptive immune systems. The current review summarizes and synthesizes the extant literature on the immune system during pregnancy, as well as the sparse existing evidence highlighting the associations between inflammation and mood, anxiety, and fear-related disorders in pregnancy. In general, pregnant persons demonstrate lower baseline levels of systemic inflammation, but respond strongly when presented with an immune challenge. Stress and trauma exposure may therefore result in strong inflammatory responses in pregnant persons that increases risk for adverse behavioral health outcomes. Overall, the existing literature suggests that stress, trauma exposure, and stress-related psychopathology are associated with higher levels of systemic inflammation in pregnant persons, but highlight the need for further investigation as the existing data are equivocal and vary based on which specific immune markers are impacted. Better understanding of the psychoimmunology of pregnancy is necessary to reduce burden of prenatal mental illness, increase the likelihood of a successful pregnancy, and reduce the intergenerational impacts of prenatal stress-related mental health disorders.

## Introduction

Stress-related psychopathological disorders like depression, anxiety, and posttraumatic stress disorder (PTSD) are heterogenous disorders characterized by negative thoughts, loss of interest and/or pleasure, fatigue, and/or hypervigilance (1). Although many studies have focused on mechanisms of risk for stress-related disorders during the post-partum period when there are significant alterations in immune function (2), significantly fewer studies have focused on understanding mechanisms of risk for stress-related disorders during pregnancy itself. Pregnancy is also characterized by incremental changes in the immune system (3), and evidence indicates that pregnant persons are also at increased risk for depression, anxiety, and PTSD (4–11). In addition to directly impacting prenatal mental health, stress-related disorders during pregnancy can result in epigenetic alterations in offspring (12) and are associated with greater parenting stress (13), suggesting these disorders can have important implications for development of psychopathology and other adverse health outcomes in offspring. It is therefore vital to understand biological mechanisms underlying increased risk for stress-related mood, anxiety, and fear-related disorders in pregnancy to inform targeted interventions to attenuate prenatal and intergenerational risk.

One biological mechanism that confers increased risk for stress-related mental health conditions but has not been well studied in the context of prenatal mental health is the immune system (14–16). Inflammation in the periphery (typically measured by pro-inflammatory cytokines and acute phase reactants like C-Reactive Protein [CRP]) is implicated in several psychiatric illnesses in the general population including depression, anxiety, and PTSD (14–16). Both endogenous and exogenous (i.e., vaccination, interferon-alpha [IFN-α] treatment, endotoxin challenge) immune challenges induce symptoms of depression and anxiety (14–16). Importantly, increased systemic inflammation in the periphery can impact the brain: inflammatory cytokines can cross through leaky regions of the blood brain barrier, be actively transported across the blood brain barrier, activate macrophages lining the brain to produce their own inflammatory cytokines, and activate cytokine receptors on the vagus nerve (14). In the brain, inflammation reduces availability of monoamines and increases extrasynaptic glutamate (which could cause excitotoxicity) and impacts the activity and functional connectivity of brain regions that are implicated in mood, anxiety, and fear-related disorders, such as the amygdala, insula, and anterior cingulate cortex (14–16). Despite established causal relationship between inflammation and mood, anxiety, and fear-related disorders, very few studies have examined how pregnancy-related changes in the immune system may contribute to symptoms of these disorders in pregnant persons. The current review summarizes and synthesizes the current literature on the immune system during pregnancy, as well as the sparse existing evidence highlighting the associations between inflammation and mood and anxiety disorders in pregnancy. We also note that there are no studies assessing the relationship between inflammation and PTSD symptoms in pregnancy. Finally, we also discuss gaps in knowledge and the importance of further understanding how immune changes during pregnancy impact prenatal mental health.

## Pregnancy and the Immune System

Pregnancy is a period of complex and profound immunological change at the fetal- parental interface (17, 18) and in the pregnant individual's periphery (3). Overall, pregnancy is characterized by a shift in balance toward the non-specific innate (e.g., neutrophils, monocytes, and natural killer [NK] cells) immune system over the acquired and antigen specific adaptive (e.g., T cells and B cells) immune system (19, 20). During the first trimester of pregnancy, a pro-inflammatory state is conferred by greater activity of the innate immune system to promote uterine binding and to establish fetal-parental vasculature (21). This pro-inflammatory state is thought to be at least partially responsible for the morning sickness symptoms experienced by many pregnant persons in the first trimester (21).

The second and third trimesters of pregnancy are characterized by shifts in the innate and adaptive immune systems (20) that results in an anti-inflammatory bias, which is characterized by the release of fewer pro-inflammatory cytokines and greater anti-inflammatory mediators (21). During these late trimesters, there is an increased ratio of M2 macrophages (that produce anti-inflammatory signals) over M1 macrophages (that produce Th1-type responses) at the parental-fetal interface (22, 23). Additionally, T helper (Th) cells in the adaptive immune system shift from pro-inflammatory Th1 cells to anti-inflammatory Th2 cells, while the balance of NK cells in the innate immune system shifts toward an NK2 cell bias over NK1 cells, which are characterized by the interleukin-18 (IL-18) receptor (24). As a result of low levels of circulating IL-18, this shift in NK balance toward fewer NK1 cells typically results in reduced concentrations of the pro-inflammatory cytokine interferon-γ (IFN-γ) (25) and tumor-necrosis factor alpha (TNFα) (26). Furthermore, monocytes in pregnant individuals also produce lower concentrations of pro-inflammatory cytokines, such as IL-18, TNFα, and interleukin-6 (IL-6), under low levels of monocyte stimulation (i.e., by a pathogen such as *E. coli* or lipopolysaccharide (LPS)) than monocytes in non-pregnant persons after stimulation (3, 22, 25, 26). Overall, the balance of M2/M1 macrophages under conditions of low monocyte stimulation is thought to be important in preventing rejection of the fetus and in maintaining a healthy pregnancy (22, 23). Concentrations of pro-inflammatory cytokines typically remain low until the end of the third trimester, when an increased pro-inflammatory state [induced by high concentrations of corticotropin-releasing hormone, CRH (27)] is thought to promote uterine contraction and expulsion of the fetus (20, 21).

Under conditions of high monocyte stimulation, monocytes produce greater concentrations of multiple cytokines than under conditions of low to no stimulation. One cytokine that is increased under conditions of high monocyte stimulation is IL-18, which activates the IL-18 receptor on NK1 cells (24), resulting in increased concentrations of IFN-γ by NK1 cells (25). Importantly, when monocytes in pregnant persons are stimulated in the presence of IFN-γ, they produce increased amounts of pro-inflammatory cytokines as compared to monocytes in non-pregnant persons stimulated under the same conditions (3, 22). In addition, IFN-γ can induce monocytes to differentiate into M1 over M2 macrophages (23). Thus, initially higher stimulation of monocytes can result in an exaggerated pro-inflammatory response and altered decidual M2/M1 macrophage balance in pregnant persons (25). This adaptation of the immune system during the second and third trimester of pregnancy provides pregnant persons with the ability to respond to sufficiently inflammatory threats, even while in a general state of reduced systemic inflammation.

The baseline anti-inflammatory bias for greater basal levels of anti-inflammatory mediators in circulation compared to pro-inflammatory signals but simultaneous exaggerated pro-inflammatory response to high levels of immune stimuli in late pregnancy likely contributes to the equivocal nature of existing data describing alterations in systemic inflammation throughout pregnancy (21). For example, one study reported wide variability in the magnitude and direction of changes in concentrations of CRP between individuals; some individuals showed a decrease in IL-6 and CRP through pregnancy, while others showed an increase (28). Additionally, another study found that concentrations of the pro-inflammatory cytokine IL-6 generally decreased across pregnancy, but showed large variability in concentrations of IL-6 at each trimester of pregnancy (29). While several studies on the modulation of the immune system during pregnancy have focused on the effects of infection or fetal trophoblast particles (25, 30), other factors that stimulate monocytes may also result in pro-inflammatory monocyte activation in pregnant persons, which may help account for the equivocal nature of data on the immune system in pregnancy to date.

## Stress/Trauma Impact Sympathetic, neuroendocrine and Immune Interactions in Pregnancy

**Figure 1:**
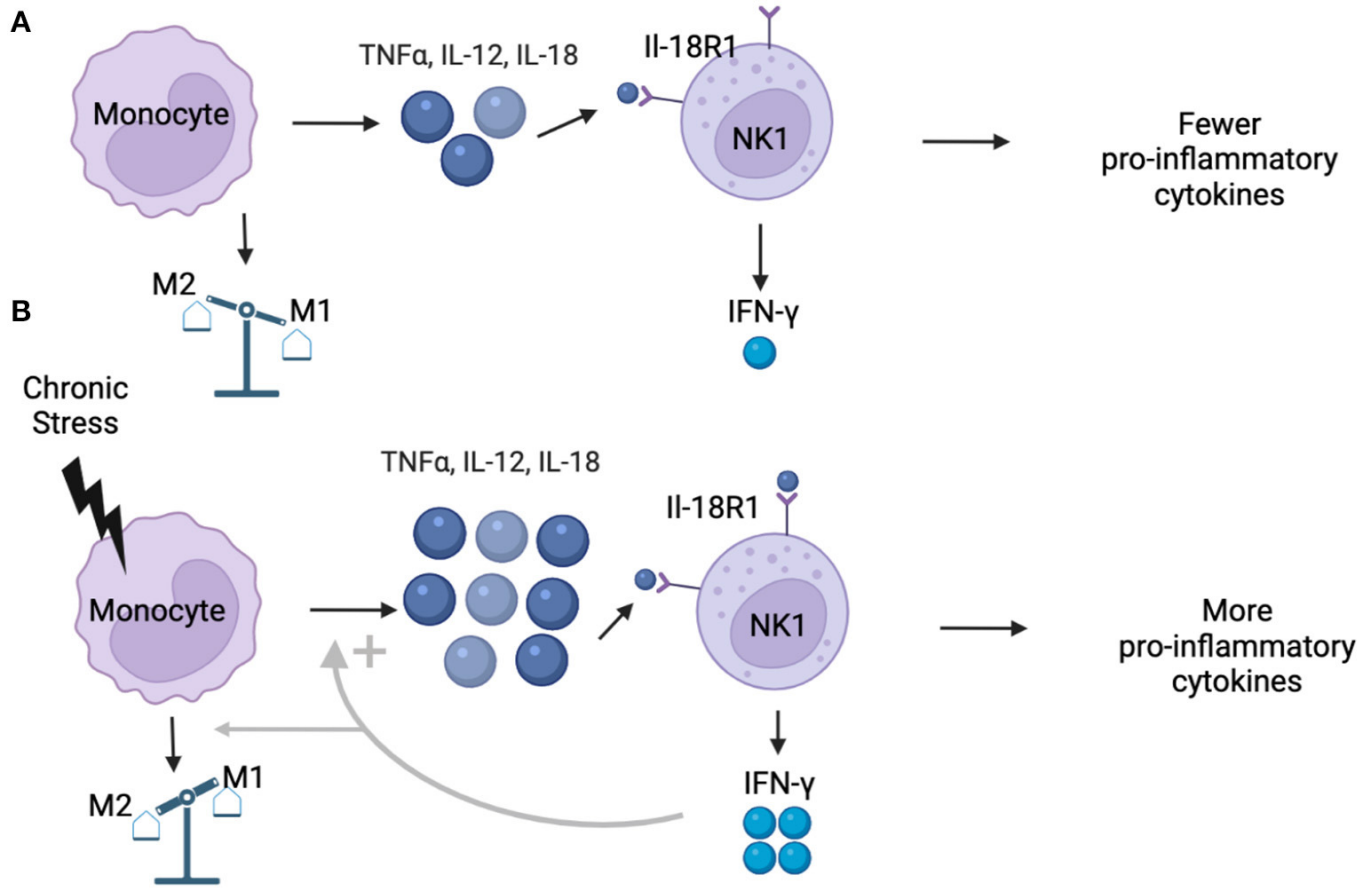
**(A)** Under normal conditions, monocytes produce low concentrations of pro-inflammatory cytokines including TNFα, IL-12, and IL-18. Simultaneously, pregnancy is characterized by shift that reduces the proportion of NK1 (IL-18R1) cells in circulation. Existing NK1 cells are weakly stimulated by low concentrations of IL-18, resulting in low production of IFN-γ, ultimately resulting in more M2 decidual macrophages and a less pro-inflammatory environment that characterizes the second and third trimesters of healthy pregnancies. **(B)** Under conditions of chronic stress, monocytes are stimulated at higher rates, resulting in greater production of pro-inflammatory cytokines, including IL-18. Higher concentrations of IL-18 activate NK1 cells at higher rates than under healthy conditions, leading to increased production of IFN-γ, which can promote an M1 over M2 bias in the decidua. When monocytes in pregnant individuals are stimulated in the presence of IFN-γ, the pro-inflammatory response is exaggerated, leading to even higher concentrations of pro-inflammatory cytokines, which could result in a pro-inflammatory bias in individuals experiencing chronic stress.

One environmental factor capable of activating monocytes and facilitating a pro-inflammatory state is stress and/or trauma exposure, which contributes to increased inflammation in non-pregnant persons (14, 15). More specifically, under conditions of acute and chronic stress exposure, the sympathetic nervous system induces pro-inflammatory cytokine release from monocytes, including the release of IFN-γ, which can prime a pro-inflammatory immune response to other potential stimulations in pregnant women (25). Thus, individual differences in stress and/or trauma exposure could partially explain the wide variation in immune signaling changes reported in pregnant women to date. For instance, pregnant individuals who experience few to no stressors might show a decrease in systemic inflammation throughout pregnancy (Figure 1A), while pregnant persons who experience more stressors, such as those with mood and/or anxiety disorders, might show an increase in systemic inflammation over the course of pregnancy (Figure 1B). This stress-induced increase in systemic inflammation may exacerbate severity of mood and anxiety in pregnant individuals, which can act in a feedforward manner to further increase stress and systemic inflammation to impact mental health.

Another mechanism by which chronic stress and/or trauma exposure facilitate increased systemic inflammation is via dysregulation of the neuroendocrine hypothalamic-pituitary-adrenal (HPA) axis [for comprehensive reviews, please see (14, 31, 32)]. Under acute stress conditions glucocorticoids (GCs; i.e., cortisol in humans) released from the adrenal glands act through their receptors to inhibit inflammatory processes by interacting with the nuclear factor kappa-B complex (32). Under conditions of chronic stress, the glucocorticoid receptor (GR) can become resistant to GCs. This GC resistance can result in exacerbated inflammatory responses to stress and trauma exposure and in impaired functioning of the HPA axis (14), including diminished GC negative feedback inhibition of the HPA axis (33). Additionally, increased inflammation itself can induce GC resistance (31), contributing to a feedforward cycle of chronic stress, GC resistance, and inflammation. Thus, chronic exposure to stress and trauma may contribute to systemic alterations in pregnant persons, who also may be undergoing neuroendocrine changes that are inherent to pregnancy itself.

Placental production of CRH increases over time during pregnancy, though much of this CRH is inactive due to its binding to the CRH-binding protein (CRH-bp) (34, 35). However, in late pregnancy concentrations of CRH-bp decrease, leading to greater levels of free and active CRH (34, 35). These higher concentrations of active CRH facilitate increased parental production of adrenocorticotropic hormone (ACTH) from the pituitary, followed by increased cortisol production (34, 35). While much of the cortisol in the third trimester of pregnancy is inactive (due to increased concentrations of cortisol binding globulin [CBG]), there is an increase in free cortisol and (36, 37) a significant decrease in CBG in the late third trimester of pregnancy (37), which results in a state of hypercortisolemia (34). This increase in cortisol during late gestation may be due to diminished GC negative feedback as assessed by the dexamethasone suppression test (38). Only one study to date has assessed GR sensitivity at the receptor level during pregnancy using *ex vivo* assays and found decreased GR sensitivity in late pregnancy (39).

Overall, dysregulation of the parental HPA axis and CRH concentrations during pregnancy (due to stress) can negatively impact fetal brain structure, neurogenesis, and neurocircuitry (40), emphasizing the need to address stress/trauma exposure and stress-related psychopathology in pregnancy. Additionally, higher levels of systemic inflammation and prenatal stress exposure can also contribute to risk for negative birth outcomes like preeclampsia (41–43) and preterm birth (44, 45), which can impact infant mortality and can have long-term health consequences for offspring (46, 47). For example, depression during pregnancy is associated with shorter gestational length, poorer neurobehavioral outcomes in neonates, and increased cortisol concentrations after a stressor in 1-year old offspring (48). Prenatal concentrations of immune markers and evening cortisol in the third trimester of pregnancy are associated with infant cortisol reactivity at 1 years old, highlighting the role of antenatal immune and stress processes on infant outcomes (48).

Stress and psychopathology during pregnancy can also alter epigenetic mechanisms in the fetus, which have been associated with greater vulnerability to psychopathology and other negative health outcomes in offspring (49). For instance, prenatal exposure to famine during pregnancy has been associated with less DNA methylation of the insulin-like growth factor II (IGF2) in offspring (50), and changes in offspring DNA methylation at many regions (including regions that regulate immune system functioning) that have been associated with adulthood triglyceride levels (51). The effects of stress during pregnancy can also have transgenerational effects, as the grandchildren of persons who were exposed to violence while pregnant show altered DNA methylation in regions associated with regulating the circulatory system (52). Psychopathology before pregnancy can also impact both pregnancy and offspring outcomes; one study found that women with a history of depression (but not depression during pregnancy) showed increased levels of the immune markers IL-8, VEGF, and MCP-1 during the third trimester of pregnancy, and their children showed altered neurobehavioral responses compared to women without a history of depression (53). Thus, the need for a better understanding of the relationships between inflammation, stress/trauma exposure, and mood and anxiety disorders in pregnancy is vital for the well-being of both the pregnant individual and offspring.

## Inflammation and Stress/Trauma in Pregnancy

Relatively few studies have examined the associations between lifetime and/or current stress and/or trauma exposure and inflammation in pregnancy specifically (Table 1). One study by Coussons-Read and colleagues found that increased current life stress levels are associated with higher concentrations of IL-6 and the pro-inflammatory cytokine TNFα, and with lower concentrations of the anti-inflammatory cytokine interleukin-10 (IL-10) in a racially diverse sample (54). This finding was replicated in a primarily white sample, where current life stress was associated with increased concentrations of IL-6 in early and late pregnancy, and with lower concentrations of IL-10 in early pregnancy (55). Stress levels in the second trimester of pregnancy and low levels of social support in the third trimester predicted elevated concentrations of CRP in the third trimester of pregnancy, and higher stress levels throughout pregnancy were associated with increased production of pro-inflammatory cytokines by stimulated lymphocytes in the third trimester of pregnancy (55).

**Table 1:** Stress/Trauma exposure and systemic inflammation in pregnancy.

**Year**	**References**	**Trimester**	**Phenotype assessed**	**Inflammatory markers**	**Main findings**
2005	(35)	1, 2, & 3	Psychosocial stress	IL-10, IL-6, TNF-α	• Higher stress is associated with greater IL-6 and TNF-α • Higher stress is associated with lower IL-10 across all trimesters
2007	(36)	1, 2, & 3	Psychosocial stress	Stimulated lymphocyte production of IL-1β, IL-6, IL-10, TNF-α; serum concentrations of TNF-α, IL-6, IL-10, CRP	• Higher stress is associated with lower IL-10 in the first trimester • Higher stress is associated with higher IL-6 in the first and third trimesters No association between stress and IL-10 or IL-6 in the second trimester • Higher stress is associated with CRP in the second trimester but not the first or third trimesters • Higher average stress across all trimesters is associated with higher average CRP across all trimesters • Higher average stress across all trimesters is associated with greater lymphocyte production of IL-1β and IL-6
2009	(47)	Any	Perceived stress	Il-6, TNF-α	• Perceived stress is not associated with IL-6 or TNF-α
2011	(40)	2 & 3	Trauma exposure	IL-6, TNF-α	• Trauma exposure is associated with TNF-α at both trimesters
2018	(37)	1, 2, & 3	Acculturation	IL-6	• Higher acculturation is associated with higher IL-6 across all trimesters
2016	(38)	2	Racial discrimination	IL-1β, IL-2, IL-4, IL-6, IL-8, IL-10	• Experiences of racial discrimination is associated with higher IL-4 and IL-6, but not with any other cytokines measured
2016	(41)	2 & 3	Intimate partner violence (IPV)	IL-6, TNF-α	• History of IPV is associated with higher concentrations of TNF-α in the second trimester • History of IPV is associated with a smaller increase in IL-6 between the second and third trimesters
2016	(44)	2 & 3	Childhood abuse	CRP, IL-6	• Childhood abuse is not associated with CRP or IL-6 at either the second or third trimester in adolescents • Adolescents with high depressive symptoms and who have experienced more abuse show higher IL-6 concentrations in the second trimester
2018	(43)	1, 2, & 3	Childhood abuse	CRP, IL-6, TNF-α	• Childhood physical abuse, emotional abuse, and emotional neglect are associated with CRP concentrations throughout pregnancy, but not with IL-6 or TNF-α. • BMI mediates a relationship between physical abuse and CRP and IL-6 concentrations across pregnancy. • The relationship between emotional abuse and CRP is not significant after controlling for BMI.

In support of the association between stress exposure and increased inflammation in pregnancy, increased acculturation, which has been associated with acculturative stress (or the stressors that accompany being an ethnic minority), has been associated with increased IL-6 concentrations throughout pregnancy in a sample of Mexican-American women (56). Interestingly, Latina women tend to show better pregnancy outcomes in comparison to other groups despite factors like lower socioeconomic status (the so-called Latina paradox) (57); however, this advantage diminishes the longer an individual is in the United States (58), potentially due to acculturation stress (56). Additionally, Black pregnant women who experienced any amount of racial discrimination in their lifetime had higher concentrations of IL-6 and interleukin-4 (IL-4) in their second trimester compared to Black pregnant women who did not report any racial discrimination in their lifetime (59). In contrast, a study with Black and white pregnant women found that pregnancy did not have an effect on IL-6 concentrations after an acute stressor (the Trier Social Stress Task; TSST); Black pregnant and non-pregnant women demonstrated stronger IL-6 responses in response to the TSST compared to white women, with pregnancy itself having no observed effect on IL-6 responses (60). However, this study focused on IL-6 responsivity to an acute stressor, while to the best of our knowledge, no studies have compared the effects of chronic stress on inflammation between pregnant and non-pregnant women.

In addition to general life stress exposure, trauma history may also impact inflammation in pregnancy. One study found that trauma exposure was associated with increased concentrations of TNFα, but not with concentrations of IL-6 in the second and third trimesters of pregnancy (61). Importantly, trauma exposure was defined categorically (any exposure to trauma or not) and not continuously, and <40% of the sample had experienced a criterion A trauma, meaning the study might not have had sufficient power to identify relationships between trauma history and concentrations of IL-6 (61). A similar study found that women who had experienced intimate partner violence (IPV) saw blunted increases in IL-6 between the second and third trimesters compared to women who had not experienced IPV, though only the degree of change between trimesters and not absolute concentrations of IL-6 varied based on exposure to IPV (62). However, only 35 out of 171 total women in this study had experienced IPV, and the study did not account for severity, amount, or timing of IPV experienced, which are important factors that may impact inflammation in pregnancy (62).

Crucially, none of the above studies examined the role of childhood trauma on inflammation in pregnancy specifically, which is thought to have a particularly strong effect on both inflammation and psychopathology in adulthood, in part due to epigenetic alterations of genes involved in stress responsive systems like the HPA axis (63). One study that focused explicitly on childhood trauma exposure found that childhood sexual abuse or physical neglect was not associated with concentrations of CRP in pregnancy, but CRP was associated with childhood physical abuse, emotional abuse, and emotional neglect. (64). Although there were no direct relationships between childhood trauma types and IL-6 or TNF-α concentrations, pre-pregnancy body mass index (BMI) did mediate a relationship between physical abuse and IL-6 concentrations (64). BMI also mediated the relationship between physical abuse and CRP concentrations, suggesting that experiencing physical abuse as a child may increase the likelihood of having a higher BMI in adulthood, which could result in higher inflammation in pregnancy (64). Conversely, a study with pregnant Latina adolescents found that childhood physical, sexual, or emotional abuse was not associated with IL-6 or CRP concentrations in the second or third trimesters of pregnancy (65).

Taken together, studies to date suggest that stress/trauma exposure confers increased risk for higher inflammation in pregnant women and even altered expression of immune genes two to six years after pregnancy (66). This relationship may be due to glucocorticoid resistance. One study examining chronic stress, cortisol levels, and inflammation found that pregnant women at high risk for chronic stress exposure (women of either minority or low income status) have higher cortisol concentrations than low risk women, and the higher cortisol concentrations associated with the high risk group are not accompanied by decreased inflammation (67). Specifically, women in the low risk group demonstrate a negative relationship between average cortisol concentration and a pro-to-anti-inflammatory cytokine measure, but this negative relationship is absent in women in the high-risk group (67). Future studies are needed to assess the role of glucocorticoid resistance as a mechanism underlying increased inflammation in pregnant women experiencing high levels of stress and/or trauma exposure.

## Inflammation and Stress-Related Psychopathology in Pregnancy

While the studies reviewed thus far suggest that stress and/or trauma exposure influence inflammation in pregnancy, the relationships between stress-related psychopathology and inflammation in pregnancy are even less clear. Most studies to date have assessed the cross-sectional relationship between psychopathology and inflammation in pregnancy, assessing whether inflammation is associated with symptoms of psychopathology and vice versa. One study found that concentrations of IL-6 at approximately 15 weeks gestation were positively correlated with depressive symptoms (as measured by the Center for Epidemiologic Studies Depression Scale or CES-D) after controlling for BMI (68) (Table 2). This study included primarily low-income women, where a little over half the women had a probable diagnosis of depression (68). Pregnant women with depression also show increased concentrations of IL-6, IL-10, TNF-α, vascular endothelial growth factor (VEGF), increased diurnal cortisol secretion, increased evening cortisol secretion, and a blunted cortisol awakening response in the third trimester as compared to pregnant women without depression (48). In addition, in a sample of Hispanic women, higher depressive symptoms were associated with higher concentrations of the interleukin 1-receptor antagonist (IL-1RA) (69), which is elevated under conditions of increased inflammation (70).

**Table 2:** Stress-Related psychopathology and systemic inflammation in pregnancy.

**Year**	**References**	**Trimester**	**Phenotype assessed**	**Inflammatory markers**	**Main findings**
2007	(51)	2	Depressive symptoms	IL-1RA	• Higher depressive symptoms are associated with higher IL-1RA
2009	(47)	Any	Depressive symptoms	IL-6, TNF-α	• Depressive symptoms are associated with higher IL-6 • Depressive symptoms have a trending association with TNF-α
2010	(53)	Any	Depressive symptoms	MIF (baseline and 1 week post-influenza vaccine)	• Higher MIF one-week post-vaccine is associated with greater depressive symptoms
2011	(40)	2 & 3	Depressive symptoms, anxiety symptoms, PTSD diagnosis	IL-6, TNF-α	• Depressive symptoms are not associated with IL-6 or TNF-α at either trimester • Anxiety symptoms are not associated with IL-6 or TNF-α at either trimester • PTSD diagnosis is not associated with IL-6 or TNF-α at either trimester
2012	(50)	2	Depressive symptoms	CRP, IL-1β, IL-6, TNF-α, IL-10	• Depressive symptoms are associated with IL-6 and IL1-β, but not with CRP, TNF-α, or IL-10.• Depressive symptoms are associated with IL-6 concentrations in women with lower BMIs but not in women with higher BMIs.• Depressive symptoms are associated with higher IL-10 in women with lower BMIs but with lower IL-10 in women with higher BMIs.
2016	(44)	2 & 3	Depressive symptoms	CRP, IL-6	• Depressive symptoms in adolescents are not associated with CRP or IL-6 at either the second or third trimester. • Adolescents with high depressive symptoms and who have experienced more abuse show higher IL-6 concentrations in the second trimester.
2017	(49)	2	Depressive symptoms, overall anxiety symptoms, pregnancy anxiety	IL-5, IL-9, IL-13, IL-12, IFN-γ, IL-4, IL-6, IL-10, TNF-α	• Higher depressive symptoms are associated with higher IL-9, IL-13, IL-12, IL-5, and a higher IFN-γ/IL-4 ratio.• Higher overall anxiety symptoms are associated with higher IL-9, IL-13, and IL-12• Higher pregnancy related anxiety is associated with higher IL-12, IL-13, and IL-10. IL-6 and TNF-α are not associated with any symptoms
2018	(48)	3	Depression diagnosis	IL-1β, IL-2, IL-6, IL-8, IL-10, TNF-α, VEGF, EGF, MCP-1, CRP	• Depression is associated with higher concentrations of IL-6, IL-10, TNF-α, and VEGF.
2019	(54)	1, 2, & 3	Depressive symptoms, anxiety symptoms	23 cytokines total, including IL-6, IL-15, CCL3, C-X-C motif ligand 8 (CXCL8), and Granulocyte macrophage colony-stimulating factor (GM-CSF)	• Higher IL-15 is associated with depressive symptoms at the first and third trimesters.• Higher IL-6 and CCL3 are associated with depressive symptoms at the third trimester.• IL-6 and CCL3 decrease through pregnancy for less depressed women, but increase for more depressed women.• Higher CXCL8 is associated with anxiety symptoms in the first and third trimesters• Higher IL-6 and CCL3 is associated with anxiety symptoms at the third trimester• IL-6 increases through pregnancy for more anxious women, but not for less anxious women• GM-CSF decreased across pregnancy for less anxious women, but not for more anxious women.

In contrast, other studies have found no association between certain pro-inflammatory cytokines and depressive and anxiety symptoms. For example, in a sample of Finnish women, IL-6 and TNF-α were not associated with either depressive, overall anxiety, or pregnancy-related anxiety symptoms in the second trimester, though symptoms were associated with other cytokines including IL-9, IL-12, and IL-13 (71). Another study with a Black sample of varying socioeconomic status also found that depression scores were not associated with CRP, but were associated with concentrations of IL-6, though only in women of lower BMI (72). The lack of association between depressive symptoms and IL-6 concentration in women with higher BMI might be due to a ceiling effect due to higher BMI resulting in greater inflammation (72). Furthermore, childhood sexual, emotional, and physical abuse interact with depression to predict higher concentrations of IL-6 in the second trimester in Latina adolescents (65). Pregnant women with more depressive symptoms may also show sensitization to immune challenges, as in a study of 22 pregnant women, those in the highest tertile of depression scores had higher concentrations of the pro-inflammatory molecule macrophage migratory inhibitory factor (MIF) than women with in the lowest tertile of depression scores 1 week after receiving the influenza vaccine (73).

Psychopathology in pregnancy may also impact the expression of cytokine trajectories throughout pregnancy. In a study of primarily minority women, depressed women (defined by having a score on the Beck Depression Inventory >9) had higher concentrations of IL-6 in the third trimester of pregnancy and showed an increase in IL-6 concentrations across pregnancy (74). Women who were not depressed showed the opposite pattern and experienced a decrease in IL-6 across pregnancy (74). The same relationship between symptoms and IL-6 concentrations was also found for anxious women (defined by endorsing a score on the State Trait Anxiety Inventory <34) (74). Anxious women had higher concentrations of IL-6 in the third trimester of pregnancy and showed an increase in IL-6 concentration across pregnancy, while less anxious women exhibited a decrease in IL-6 across pregnancy (74). Importantly, relatively few participants in this study endorsed clinically significant symptoms of anxiety or depression (14% of the sample was in the depressed group and 29% in the anxiety group) (74). These effects might be stronger in samples with higher rates of psychopathology.

## Implications and Future Directions

Overall, the limited existing literature indicates that stress and/or trauma exposure and the presence of depression can impact inflammation in pregnancy. However, the equivocal natures of some studies and disparities in immune markers assessed highlight the need for future well-powered and longitudinal studies to tackle the critical gaps in knowledge that remain. Further work is necessary to better understand the relationship between psychiatric symptoms and inflammation in pregnancy, especially given the existing evidence showing increased risk for psychopathology during pregnancy (4–11). Furthermore, most studies examining associations between psychiatric symptoms and inflammation in pregnancy focus on depressive symptoms, with only a couple also including anxiety symptoms. No known studies have investigated associations between inflammation and fear-related disorders like PTSD in pregnant individuals, despite evidence of a relationship between inflammation and PTSD symptoms in non-pregnant persons (15, 75). This is an important gap that should be addressed, as previous studies show that pregnant persons are also at increased risk for PTSD and increased psychophysiological hyperarousal (8, 10). Finally, it is important to better understand how experiencing stress and adversity during pregnancy and the presence of stress-related psychopathology impacts inflammation through alterations in behavior, such as eating (76) and sleep (77).

Another limitation of existing studies characterizing the impacts of stress/trauma and psychopathology on inflammation in pregnancy, especially those on trauma exposure, consist of samples that have experienced relatively few traumatic events over their lifespan, including childhood trauma which confers disproportionate risk for adverse health outcomes in adulthood (78). It is especially important to understand the relationships between stress/trauma exposure and inflammation in pregnancy in order to address health inequities in pregnancy-related adverse health outcomes that may be driven by increased systemic inflammation. For example, Black individuals are disproportionately affected by preterm birth and preeclampsia, even after accounting for education and socioeconomic status (79, 80). Importantly, studies suggest Black women have higher concentrations of IL-6 in the second and third trimesters of pregnancy compared to non-Black women (81). Furthermore, Black women show greater IL-6 release in response to LPS stimulation of leukocytes across pregnancy, as well as greater glucocorticoid resistance, compared to white women (82).

The health inequities that Black pregnant persons face are not due to Black race itself, but to high rates of stress and trauma exposure (83) and experiences of racism (84). Black women are exposed to disproportionate rates of trauma (85), and also experience racism at the systemic level and individual racial discrimination (86, 87). Crucially, experiencing racism has been linked to poor pregnancy outcomes like preterm birth (88–90). Racial discrimination can be thought of as a chronic and unpredictable stressor (91, 92), and could therefore increase likelihood of chronic and systemic inflammation in Black persons. If, as predicted, pregnant persons are more sensitive to immune threats, it is possible that Black pregnant individuals show stronger inflammatory responses to chronic racial discrimination than when they are not pregnant. Black pregnant individuals might also be experiencing more racial discrimination while pregnant due to discrimination through the health care system as they receive prenatal care (87). Taken together, it is possible that experiencing racism might be responsible for health inequities in preterm birth by increasing inflammation. Thus, a better understanding of the effects of stress and trauma on inflammation in pregnancy is critical to address pregnancy health inequities experienced by Black individuals and inform intervention strategies to mitigate prenatal and intergenerational risk.

In conclusion, most existing studies on the relationship between stress/trauma, stress-related mood and anxiety disorders, and inflammation in pregnant persons are cross-sectional and thus unable to establish directionality of the relationship; some studies suggest that increased inflammation leads to more anxiety or depression, while other suggest that higher mood or anxiety symptoms alter inflammation. Although it is not feasible to directly manipulate stress/trauma exposure and immune functioning in pregnant persons, future studies could focus on mood, anxiety, and PTSD symptoms before and after pregnant individuals receive treatments impacting the immune system like vaccines, as has been done in the non-pregnant population and in pregnant persons (73). Ultimately, research on the relationships between stress/trauma, inflammation, and mood, anxiety and PTSD symptoms could help explain the wide variability in immune system functioning seen in pregnant persons and identify those most at-risk for adverse behavioral health outcomes over the course of pregnancy. This research could allow for early identification of pregnant individuals at increased risk for abnormally high levels of inflammation, allowing for preventative, targeted care to increase likelihood of a healthy pregnancy and healthy offspring.

## Author Contributions

MR and VM conceptualized this review. MR and BB performed the literature search. MR completed the writing of the first draft of the review. BB and VM contributed to final version. All authors have approved its final form.

## Funding

This work was supported by the National Institute of Health (MH115174 to VM).

## Conflict of Interest

The authors declare that the research was conducted in the absence of any commercial or financial relationships that could be construed as a potential conflict of interest.

## Publisher's Note

All claims expressed in this article are solely those of the authors and do not necessarily represent those of their affiliated organizations, or those of the publisher, the editors and the reviewers. Any product that may be evaluated in this article, or claim that may be made by its manufacturer, is not guaranteed or endorsed by the publisher.
